# Sensor-Based Predictive Maintenance with Reduction of False Alarms—A Case Study in Heavy Industry

**DOI:** 10.3390/s22010226

**Published:** 2021-12-29

**Authors:** Marek Hermansa, Michał Kozielski, Marcin Michalak, Krzysztof Szczyrba, Łukasz Wróbel, Marek Sikora

**Affiliations:** 1 Department of Computer Networks and Systems, Silesian University of Technology, ul. Akademicka 16, 44-100 Gliwice, Poland; marek.hermansa@polsl.pl (M.H.); michal.kozielski@polsl.pl (M.K.); marcin.michalak@polsl.pl (M.M.); lukasz.wrobel@polsl.pl (Ł.W.); 2 Somar S.A., ul. Karoliny 4, 40-186 Katowice, Poland; K.Szczyrba@Somar.com.pl

**Keywords:** outlier detection, XAI, false positive reduction, multidimesional timeseries analysis, vibration measurements, predictive maintenance

## Abstract

In this paper, the problem of the identification of undesirable events is discussed. Such events can be poorly represented in the historical data, and it is predominantly impossible to learn from past examples. The discussed issue is considered in the work in the context of two use cases in which vibration and temperature measurements collected by wireless sensors are analysed. These use cases include crushers at a coal-fired power plant and gantries in a steelworks converter. The awareness, resulting from the cooperation with industry, of the need for a system that works in cold start conditions and does not flood the machine operator with alarms was the motivation for proposing a new predictive maintenance method. The proposed solution is based on the methods of outlier identification. These methods are applied to the collected data that was transformed into a multidimensional feature vector. The novelty of the proposed solution stems from the creation of a methodology for the reduction of false positive alarms, which was applied to a system identifying undesirable events. This methodology is based on the adaptation of the system to the analysed data, the interaction with the dispatcher, and the use of the XAI (eXplainable Artificial Intelligence) method. The experiments performed on several data sets showed that the proposed method reduced false alarms by 90.25% on average in relation to the performance of the stand-alone outlier detection method. The obtained results allowed for the implementation of the developed method to a system operating in a real industrial facility. The conducted research may be valuable for systems with a cold start problem where frequent alarms can lead to discouragement and disregard for the system by the user.

## 1. Introduction

The widespread use of sensors in industry allows for data acquisition, which, combined with advanced methods of analysis, can significantly improve and optimise production. Therefore, the data can be used not only to monitor the current state of the process and devices, but also to predict this state. The application of predictive methods to sensory data representing production process, including machine condition, allows for early identification and prediction of the faulty or hazardous process state [1] or machine break down [2,3]. Preventing industry equipment failures, known as Predictive Maintenance (PdM), has developed over years and currently uses a variety of data-driven analytical methods.

Application of classification and regression methods [4] is possible when there is a sufficient representation of failures in historical data. Only such a representation will allow the use of machine learning methods to generate a model that can predict machine breakdown in the future. As maintenance services try to keep the machines in good condition, the problem is that the number of events describing a machine failure may be small or even zero. In such a case, it is not possible to generate a classifier, however, outlier detection methods can be applied to identify measurements representing the upcoming failure state of the machine.

The motivation for this work is related to the analysis of two use cases that are industry-based. The first issue is related to the process of coal crushing, which is applied in a coal-fired power plant. In this case, the monitored machine is a crusher that prepares the fuel for feeding to the boiler. The second issue is related to the process of a raw material transportation for a converter steel plant in a foundry. In this case, the monitored machine is a gantry being one of the key elements of the production line. In the case of both of these industrial machines, the analysis was based on vibration and temperature measurements that were collected. Furthermore, in both cases, the collected measurements contain too few examples representing a failure to generate a classifier that allows failure prediction. This shows that in some cases, prediction of machine failure can not be based on the supervised machine learning approach. Therefore, a system is required that is able to identify undesirable events without knowing historical examples and report them as alarms. However, there is a risk that the operator responsible for the maintenance of the process and the condition of the machines will be flooded with the number of events and will completely stop paying attention to the tool that was intended for support.

The aim of this study is to present a data-driven method analysing sensory data to predict machine failure or identify its deteriorating condition, so that users can plan maintenance work and avoid unplanned downtime. This method was prepared based on real-life data with the assumption that the created solution will be used in industry as a result of the implemented project.

The goal of the method is to operate on sensor data transformed into a multidimensional feature vector and, as in classic SCADA systems, to report events and wait for their handling. The method is adaptive, and it utilises a sliding window to analyse the consecutive batches of data. Additionally, the method is interactive—it takes into account the operator’s justification of the current machine state and utilises it in further operation. Finally, the method uses eXplainable Artificial Intelligence (XAI) solutions to explain what influenced the recommendation of an alarm. Interactivity and explainability are designed in the system to reduce the number of false positive alarms.

The contribution of this work includes:

- Data-driven, adaptive, and interaction-oriented method to identify undesirable events where such events are underrepresented,
- wWrkflow with particular emphasis on data preprocessing, anomaly identification, and process optimisation,
- Application of explainable artificial intelligence methods to outlier identification,
- Comprehensive presentation of both the system performing the identification task and its operation within the presented case studies.

The paper is organised as follows. Section 2 presents an overview of previous research related to the presented topics. Section 3 describes the proposed method, its assumptions, components, and the proposed measures of the method evaluation. Section 4 presents the industrial facilities whose monitoring motivated the research presented and for which experimental studies were carried out. Section 5 presents the experiments conducted to evaluate the proposed method on both collected real data sets and the synthetic data set. Section 6 summarises the research presented.

## 2. Related Works

The paper refers to application of outlier analysis of stream sensor data coming from crushers and gantries. In this section, a brief review of outlier detection methods and PdM applications will be presented.

### 2.1. Outlier Analysis

Outlier analysis—also known as anomaly detection—is one of the popular data analysis goals. It assumes that there exist typical and non–typical observations in the data. Even if such assumptions are quite intuitive, it is quite hard to provide a proper and strict definition of the anomaly. One of the oldest definitions [5] claims that “an outlying observation is one that appears to deviate markedly from other members of the sample in which it occurs”. Several years later [6], a different approach was presented—an outlier is an observation that deviates so much from the other observations so as to arouse suspicions that it was generated by a different mechanism. In the work [7], an outlier is defined as the observation that is inconsistent with the rest of data. Such an approach was later [8] explained in more detail: An outlier is the observation that does not follow the same model as the rest of the data.

In the paper [9], three different anomaly type divisions are provided:

- due to their range:
  – Point—only single observation is considered to be an outlier,
  – Collective—the group of observations similar to each other is considered to be outliers as all of them behave differently from other data;
- Due to their scope:
  – Local—the observation generally differs from its neighbors, the difference from other data is not so significant,
  – Global—the observation differs from all other data;
- Due to the type of the input data:
  – Vector—similarity of objects is calculated on the basis of their space location (e.g., the Euclidean distance between vectors),
  – Graph—similarity of objects depends on their graph location.

Moreover, in the case of time series (or the stream data) analysis, it is also worth mentioning the context outlier. The same vector may be interpreted differently due to its location on the time scale. For example, considering the average daily temperature in Poland, the value 18 (in °C) may be not surprising in May, however, it should be definitely interpreted as an anomaly in December.

Through the decades, dozens of outlier detection methods have been developed. Some of them represent the statistical approach starting from the well–known 3σ criterion, Grubb’s test [7], or GESD (Generalized Extreme Studentized Deviate Test) [10]. Other ones take into consideration the local data dispersion like LOF (Local Outlier Factor) [11] and RKOF (Robust Kernel–Based Local Outlier Factor) [12]. In addition, clustering techniques are applied for such an issue, like DBSCAN (Density–Based Spatial Clustering of Applications with Noise) [13]—the main idea is to interpret the noise (samples not assigned to any cluster) as outliers. Another anomaly detection technique that uses data partitioning (however, built with completely different assumptions than in case of DBSCAN) is Isolation forest [14]. This method represents the identified partitioning as a binary tree, where the longer the path to the object, the more typical the object is. Observations lying close to the tree root are considered to be anomalies. Classification algorithms are another group of methods that are used for anomaly detection. Support Vector Machine (SVM) [15] was successfully modified for anomaly detection—the one-class SVM algorithm [16] identifies the optimal margin between the typical data and the “noise”. Besides, regression methods (CART in [17]) may become useful for context outlier detection. In recent years, deep neural networks with autoencoder architecture, such as LSTM networks [18] were applied to identify outliers [19]; methods exploit the fact that outlier examples are characterised by a high reconstruction error as result of autoencoder application.

### 2.2. Predictive Maintenance (PdM)

Predictive Maintenance plays a very important role in a modern multiple type device operation. In the literature, one may find many fields of its application like railway transport [20], power industry [21], or even marine industry [22]. The main idea of PdM is to provide an analytical tool for analysing a current diagnostic state of the machine/system and predict the estimated time to its failure.

Among many applications of PdM in the industry, it is worth focusing on anomaly detection approaches. In the paper [23], several methods of anomaly detection [24,25,26] were used for welding process data analysis. The other work [27] presents the artificial neural networks application for anomaly detection in photovoltaic systems. The neural networks were also used for anomaly detection in compressed air generation systems energy consumption [28]. Moreover, in the paper [29] one may find the joined use of XAI and LSTM models for detection of anomalies in data describing the hot-rolling process.

### 2.3. Vibration Level-Based PdM

The level of selected elements vibration is a very popular input signal for a device diagnostic state estimation. Many of them may be found in the industry. The majority of them applies the Fourier transform [30,31,32,33] or wavelet transform [34,35,36] of the original signal as a preprocessing step. However, the above mentioned techniques require continuous data acquisition and analysis. Such an approach could not be considered in the case presented in this paper – it was assumed that the sensor collects data and broadcasts it only in well-defined moments to assure the very low-level of power consumption.

In the context of vibration-based predictive maintenance, data fusion has recently become a more frequently addressed issue. Various studies show how different data fusion approaches [37,38,39,40] can be used and how they affect the fault diagnosis of gearboxes and rotating machinery in general. Research on the application of data fusion methods for the approach presented in this work is one of the possible directions for future work.

To summarise the overview of the field of the proposed approach, most of the work on predictive maintenance focuses on the use of machine learning methods, which require an accessible representation of failures in the data. Furthermore, there is a lack of work presenting how the number of false positive alarms can be reduced, improving the perception of the system by the machine operator and how to use the operator experience in the method. It follows that there is a need for the proposed method. The method that is comprehensive, dedicated to the cold start problem, taking into account the machine operator and proving its quality on real industrial data.

## 3. Proposed Method

The proposed method outlined below was inspired by the needs identified in the industrial solutions analysed. The method aims to identify machine states that may be alarming and may indicate impending failure. At the same time, to be useful, the method tries to limit the number of alarms presented to the operator.

The presentation of the method consists of an overview of its successive steps, a description of the assumptions made for the method, and an extended description of the selected components of the method.

The proposed method consists of two phases. The first phase is to initialise the method and at this stage, a training set of measurements is collected. Next, the data creating the training set is processed—in particular, aggregation and feature extraction are performed. Training data transformed in this way are used to generate a model that performs the task of outlier identification, as outliers of measurements may indicate a changing state of the device. Finally, an explanatory model based on the XAI method is created for the constructed solution.

**Figure 1:**
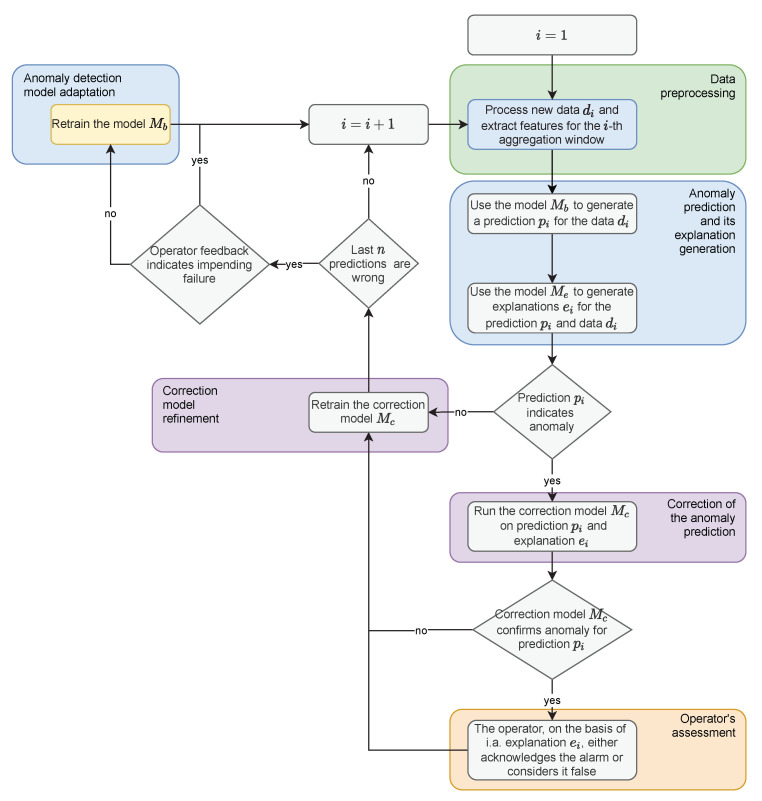
Diagram of the main phase of the proposed method.

The second main phase of the method is performed in a loop. Constant incoming measurements are collected at equal intervals defining the frame for data processing and aggregation. The data prepared in this way is delivered to the model generated in the initialisation phase, which performs the prediction. Prediction is followed by an explanation of the model decision. If an outlier is identified by the model, the correction model is run. The purpose of the correction model is to limit the number of alarms reported to the operator. If the correction model confirms the deviation from the norm, the result is presented to the operator as a potential alarm. The operator then confirms the alarm or considers it false on the basis of, among other things, the explanation provided. After each observation state is established, the correction model is refined. As a consequence, the operator receives fewer false positives in the future. If a specific proportion of recent observations is incorrectly labeled as false positives during the run of the process, the base model is re-trained. The described steps of the method are shown in Figure 1 in the form of a block diagram. For the sake of simplicity, the initialisation of the correction model, which is carried out only when a sufficient number of false alarms is reported, which may constitute the training set, was omitted on the diagram.

The proposed method is based on several assumptions which are listed below.

- The launch of the method is preceded by the period of collecting data that can be used to initiate the method (to create the first model). This period does not have to include positive examples (failures or repairs).
- It is assumed that the period preceding the failure is characterised by changes in the operation of the device. The purpose of the proposed system is to identify such changes in this period. The length of the period is a parameter of the method.
- In the period preceding the device failure, the model is expected to mark all observations as outliers. Apart from this period, the share of outliers is assumed to be very small and is a parameter of the method.
- The period immediately following the device renovation was assumed to be the time needed for the device components to run in. During this time, the model is not launched or re-trained. The length of this period is a parameter of the method. After this time the work characteristics are expected to normalise.
- It is assumed that the operator is able to determine the approximate condition of the device on the basis of other signals or on-site analysis. Therefore they are able to confirm or reject an alarm reported by the system.

The three components of the method that should be discussed in more detail are data preprocessing process, operation of the models creating the proposed method, and the approach to evaluation of its performance that was used in the experiments.

### 3.1. Data Preprocessing

The analysed data has the form of a multidimensional time series and it concerns information about the recorded vibration values—maximum and effective value and temperature. The data preparation process is performed in the steps presented below.

The first step is to determine the scope of the aggregation. Data is aggregated based on a sliding window. The size of the window is a parameter of the method. Its beginning and end have to be fixed in relation to the shift cycle in order to aggregate data from the same periods during the day. Therefore, the window size is a divisor of 24 and is expressed in hours.

The next step of data preprocessing is to remove duplicates. Duplicate values result from the fact that on the monitored object, one sensor may be within the range of more than one data collecting station. Thus, data from one sensor can be stored in the database by, for example, two stations.

Preprocessing is continued with normalisation of the sampling rate. Sensors within the facility record data at the same time intervals, but not necessarily at the same time. Hence, it is necessary to harmonise the time stamps for all sensors. In addition, the intervals between measurements within the sensors may vary due to missing data or the specificity of the sensor operation.

Within the next step of data preprocessing, missing values are managed. If the interruption in data transmission causing the missing values is short enough, it is assumed that the value of the last measurement may be repeated. Otherwise, examples representing missing data are saved in the collected data. The value of the threshold is a parameter of the method.

The next step in preprocessing the data is to remove periods of inactivity of the device from the data set. The basis for the identification of idle periods are measurements for which the registered effective value of vibrations does not exceed the assumed threshold. This value has to be determined empirically and may be different for each object and sensor. Additionally, there are deleted selected values exceeding the defined threshold, but representing the return of the operating characteristics to the operating mode or a gradual fading out.

Finally, for each of the extracted aggregation windows, a number of parameters are calculated in order to describe the characteristics of the variables.

### 3.2. Models Included in the Method

The proposed solution provides for the operation of two models. The base model is created on the basis of the collected measurement data. The purpose of this model is to identify outliers in the data in order to alert the operator to an abnormal condition of the device. This model can be generated from recently collected examples, where the size of the learning data window is a model parameter. Data preceding all past failures or maintenance incidents can also be used to generate the model. The quality of the base model is controlled and it is adjusted to the new data if the number of identified outliers and thus the number of reported alarms exceeds an assumed threshold value. This ensures that the model does not flood the operator with alarms.

The second model used within the proposed method is the correction model. The task of this model is to verify the outlier identification information. This model classifies whether a given outlier identification alert should be presented to the user. The learning data for this model contains the same attributes as the learning data for the base model. However, the model learns the alarm relevance decisions made by the operator. The model is adapted to the new data after each example is accepted or rejected by the operator. The correction model is run provided that a sufficient number of observations have been collected, in particular those that have been incorrectly flagged as outliers by the base model. If the training set is unbalanced, the observations are given appropriate weights. The operator decides whether to accept an identified observation as an outlier or to reject it based on their own experience and on the explanations provided by the XAI method.

### 3.3. Evaluation Approach

Evaluation of algorithms that perform the task of outlier identification can be performed using such measures such as: Precision and recall [41] or rank-power [42]. However, precision and recall are insufficient to fully capture the algorithm’s effectiveness, especially when comparing algorithms that result in a different number of anomalies. In particular, precision can take a low value because the number of potential anomalies identified is large. The rank-power measure is resistant to the above problem, however, not every algorithm allows for ordering the results according to the certainty of the decision made, which limits the use of this approach. Therefore, in this study three new measures: *N*, *P*, and Score were introduced to evaluate the quality of the proposed system.

Given the *D* data set, assume that the outlier detection algorithm identifies m>0 potential anomalies, of which $m_{t}\left(\leq m\right)$ are true outliers. If *D* includes $d_{t}\left(\geq m_{t}\right)$ true outliers, *P* is defined as:

(1) $$P=\frac{m_{t}}{d_{t}}.$$

*P* is equal to 1.0 if all true outliers are found by the algorithm. *N* is defined as:

(2) $$N=\frac{m-m_{t}}{D-d_{t}}=\frac{m_{f}}{d_{f}},$$

where $m_{f}\left(\leq m\right)$ is the number of normal values marked incorrectly as outliers and $d_{f}$ is the number of all normal (non-outliers) values. *N* is equal to 0.0 if the algorithm does not mark any normal value as an outlier. Score combines the two measures presented above into a single value and it is defined as:

(3) $$Score=\sqrt{(1-N)\cdot P}.$$

It equals 1.0 when all the outliers were identified as outliers and all the normal values were identified as normal.

## 4. Monitored Objects

The method proposed in this paper was motivated by case studies known from industry. The analyses presented in both case studies concern measurements performed by wireless vibration and temperature sensor WS-VT1 manufactured by Somar S.A. (https://somar.com.pl/en/temperature-and-vibrations-wireless-sensor-ws-vt1/, accessed on 30 November 2021). The measurement is performed by a transducer built using microelectromechanical systems (MEMS) technology. Acceleration is measured in three axes, over ranges of ±2 g/±4 g/±8 g/±16 g and up to 2.5 kHz. Additionally, the sensor allows for ambient temperature measurement, which is performed by a digital temperature transducer. The transducer makes measurements with a resolution of 12 bits and an accuracy of 1 °C in the range from −55 do +85 °C. Both transducers can be switched to sleep mode to save power. Measurements are managed by a 16-bit microcontroller equipped with a radio transceiver operating at 860 MHz. The entire component is powered by a 1/2 AA lithium-ion battery with 1.2 Ah capacity. In basic operation mode, the sensor is periodically awakened from sleep mode so that acceleration, temperature, and battery level can be measured. The transmission range depends on radio wave propagation conditions and is up to 500 m.

**Figure 2:**
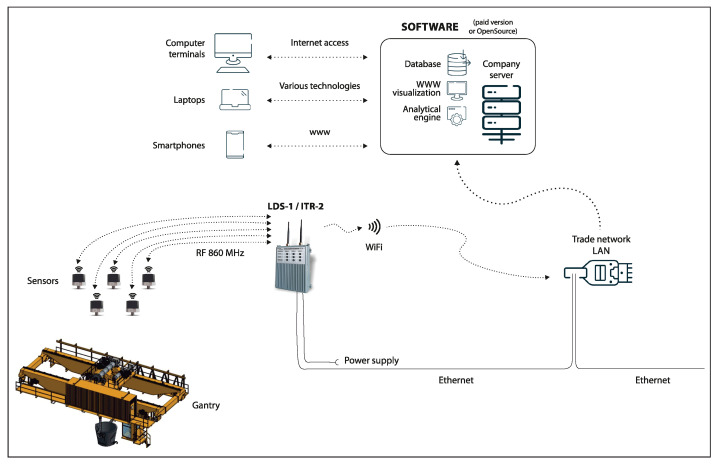
Monitoring system architecture.

The sensors are components of a monitoring system designed to acquire, monitor, and analyse the level of vibration on the equipment. The architecture of the monitoring system is schematically illustrated in Figure 2.

### 4.1. Coal Crusher

**Figure 3:**
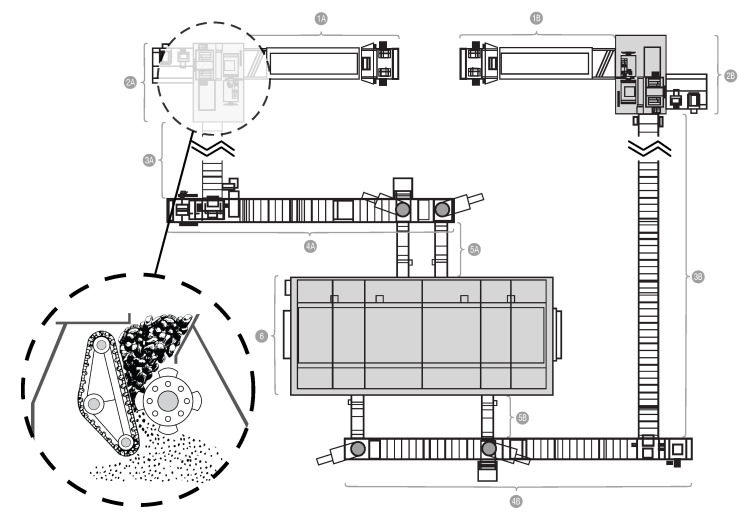
Diagram of the monitored object, which includes: Crushers (2A and 2B), feeders (1, 3, 4, and 5 (both A and B in each case)), and a boiler (6).

**Figure 4:**
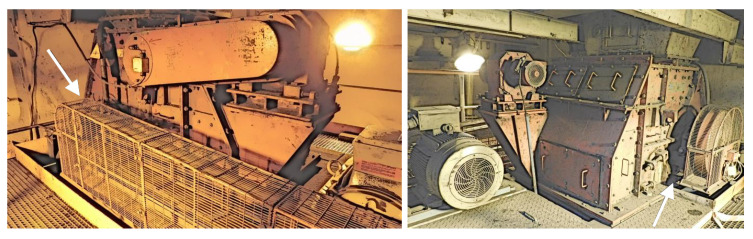
Photography of both sides of the monitored crusher—the approximate location of the sensors is indicated by arrows.

The subject of the first case study is the power unit of a coal-fired power plant. Its diagram is presented in Figure 3. The fuel, which is coal, is transported to the boiler (item 6 in Figure 3) by two routes composed of various types of feeders. In order to obtain the appropriate granulation, the fuel is crushed while passing through one of the crushers (elements 2A and 2B in Figure 3). In the crusher’s grinding chamber, coal is crushed by rows of steel hammers attached to a rotating shaft. The shaft is driven by an electric motor using a transmission belt. There is a pair of sensors collecting vibration and temperature measurements. The sensors are placed on the shaft—one on the adjustment side, the other on the drive side. The photo presenting the monitored crusher from both sides is presented in Figure 4.

The nature of the machine operation and high dust levels involved make it particularly susceptible to drive failures, and any failure requires an undesirable reduction in energy production capacity.

### 4.2. Gantry

The subject of the second case study is a transport line in a steelworks converter. The key element of the line is the gantry, whose cycle is as follows. The gantry, with a lifting capacity of 500 tonnes, takes full ladles of raw steel from the transport trolleys and transports them towards the inlet of the converter. The raw steel is poured in by tilting the ladles and then the gantry puts the empty ladles back on the trolleys.

**Figure 5:**
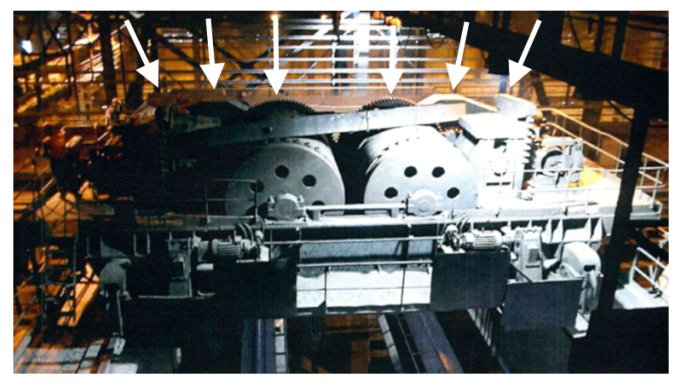
Photography of the gantry drive system—the approximate location of the sensors is indicated by arrows.

**Figure 6:**
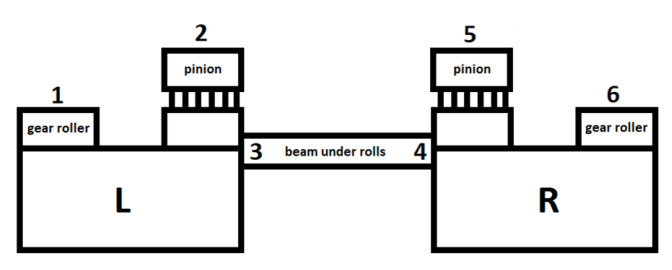
Diagram of the gantry drive system with location of vibration sensors (1 to 6).

The drive system responsible for raising and lowering the ladle is presented in Figure 5 and schematically illustrated in Figure 6. The gantry has two drives—left (L) and right (R). The drives are connected by rollers with gears that reduce the roller speed in the main lifting mechanism. The vibration sensors are mounted on the rollers, pinion bearings, and on the supporting beam as pointed in Figure 6 (locations 1 and 6, 2 and 5, and 3 and 4, respectively).

The gantry operates in difficult environmental conditions. Metal oxide dust is omnipresent in the production hall and it increases the vibration level of the entire gantry. The vibrations of the gears are transmitted to other parts of the structure. In extreme situations, operation of the gantry becomes impossible or possibly the operators are forced to reduce engine gears, which results in reduced production efficiency. In such situations, the gearbox requires a major overhaul.

## 5. Experiments

The presented solution was designed for the analysis of data from vibration and temperature sensors located in critical points of monitored objects, in particular on moving parts of machines. Each analysis concerns all sensors in the facility—the condition of the entire device is diagnosed, not its individual elements.

The evaluation of the proposed method was carried out on selected data sets. The basic assumptions of the method and its correct operation for data generated according to the assumed characteristics were verified on synthetic data. The case studies tested the performance of the method on real data collected on the coal crushers and the gantries presented in Section 4. The experiments conducted allowed not only to verify the value of the method in itself; the research verified what effect the individual parameters of the proposed solution have on the quality of the method on selected data.

The conducted research was carried out in the Python language environment [43] (version 3.7). The main libraries that were used are: Pandas [44], which provides structures for representing data sets, scikit-learn [45] which provides implementations of most of the algorithms used, tsfresh [46], which implements feature extraction and selection, and shap [47], which provides XAI methods.

### 5.1. Data Sets

**Figure A1:**
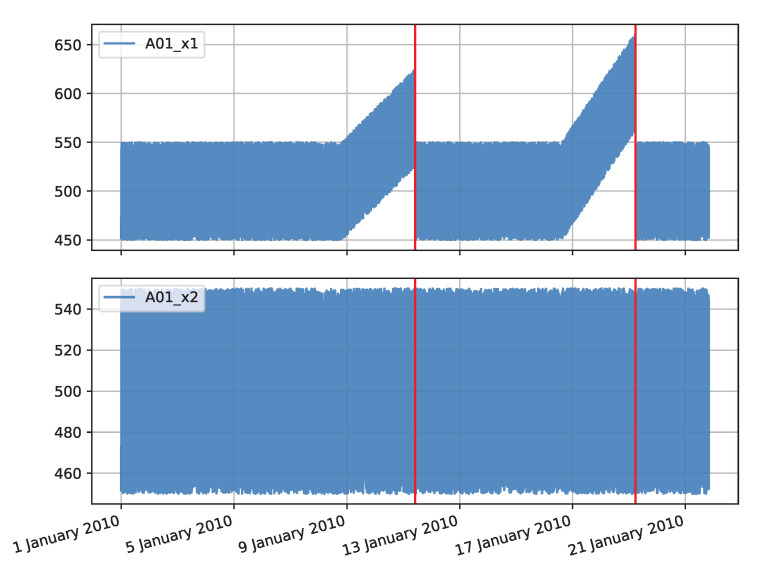
Plots of values for the synthetic data set consisting of *x*1 (red lines indicate failure locations) and *x*2 series.

The synthetic data set was generated as two time series containing 30,000 observations each. In the first time series (denoted as *x*1), the operating characteristics were periodically changed to simulate an impending failure. Two periods were generated in which the values of the simulated measurements gradually increase and then rapidly return to the regular value. In this way, the deteriorating condition of the device, which returns to normal after maintenance work, was simulated. The characteristic of the second time series (denoted as *x*2) is stable. Both time series are illustrated in Figure A1 and their characteristics are presented in Table A1, in Appendix A.

**Figure A2:**
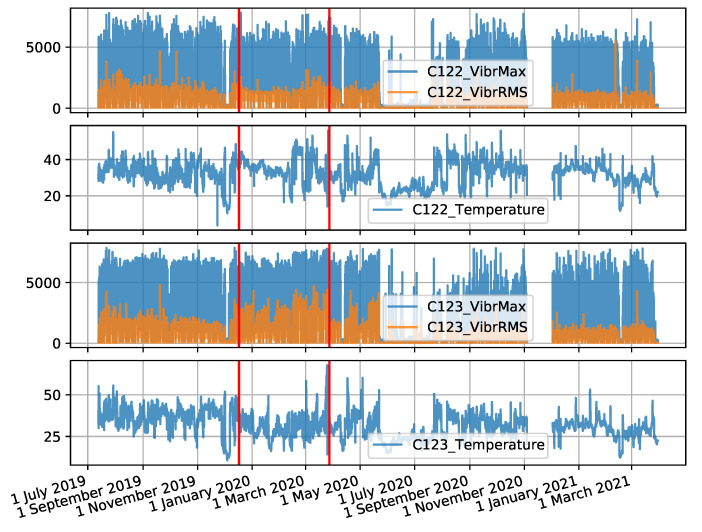
Time series for the NW-10 crusher collected by two sensors (the red colour indicates the locations of failures).

**Figure A3:**
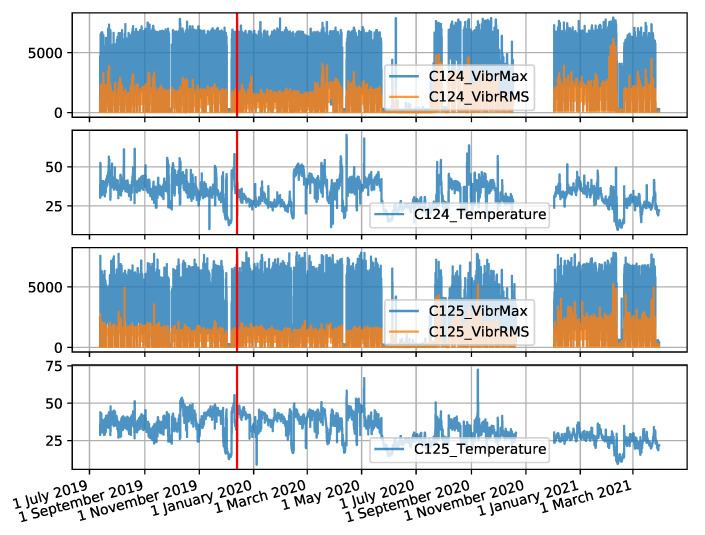
Time series for the NW-20 crusher collected by two sensors (the red colour indicates the locations of failures).

The coal crusher data set was created from measurement values recorded for two crushers operating at the same boiler of the power plant between 13 July 2019 and 30 March 2021. There are 2 sensors located on each machine. On the NW-20 crusher these are sensors C124 (drive side) and C125 (control side), and on the NW-10 crusher these are sensors C122 (drive side) and C123 (control side). The data collected includes three attributes: Maximum vibration value, root mean square (RMS) value of vibration, and temperature. The interval between observations is 10 s. The time series forming the data set are shown in Figure A2 and Figure A3, in Appendix A. The descriptive statistics of each attribute for each sensor are presented in Table A2, in Appendix A.

**Figure A4:**
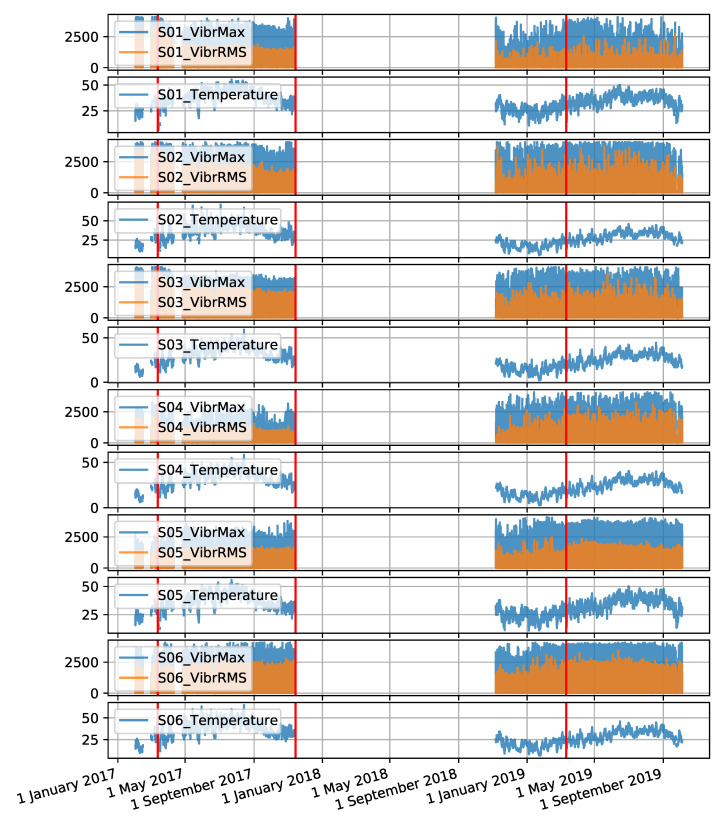
Time series for the S201 gantry collected by six sensors (the red colour indicates the locations of failures).

**Figure A5:**
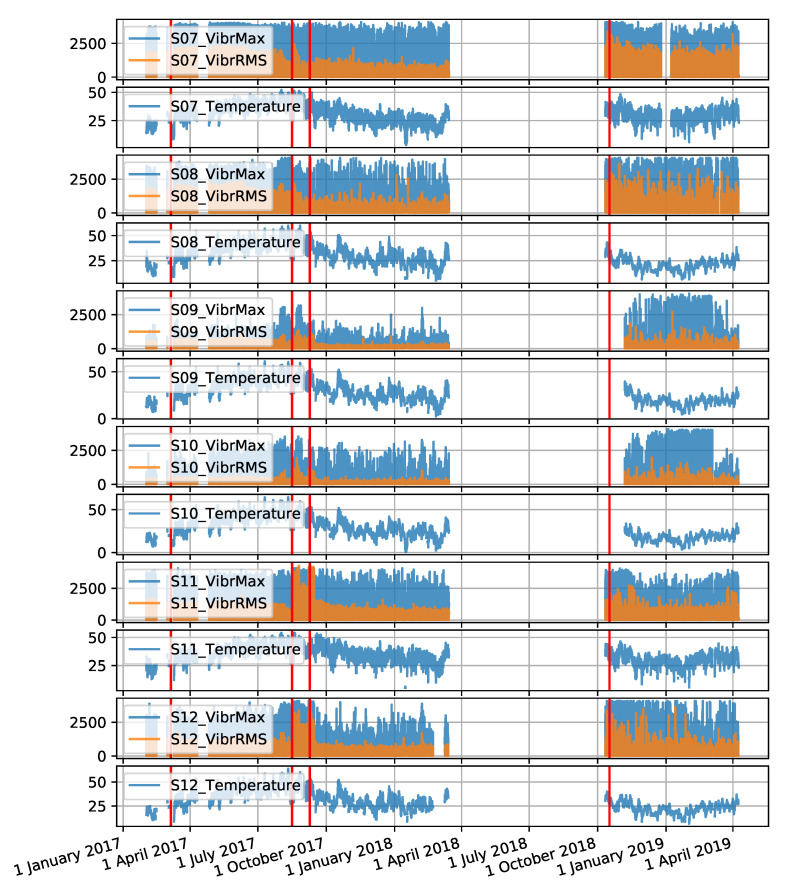
Time series for the S202 gantry collected by six sensors (the red colour indicates the locations of failures).

The gantry data set was created from measurement values recorded for two gantries named S201 and S202, over a period of 4 months. There are 6 sensors located on each machine. The sensors are denoted S01 to S06 for the S201 gantry and S07 to S12 for the S202 gantry. The data collected includes three attributes: maximum vibration value, root mean square (RMS) value of vibration and temperature. The interval between observations is 30 s. The time series forming the data set are shown in Figure A4 and Figure A5, in Appendix A. The descriptive statistics of each attribute of the data collected by sensors located on the monitored gantries are presented in Table A3, in Appendix A.

### 5.2. Experiment Settings

**Figure 7:**
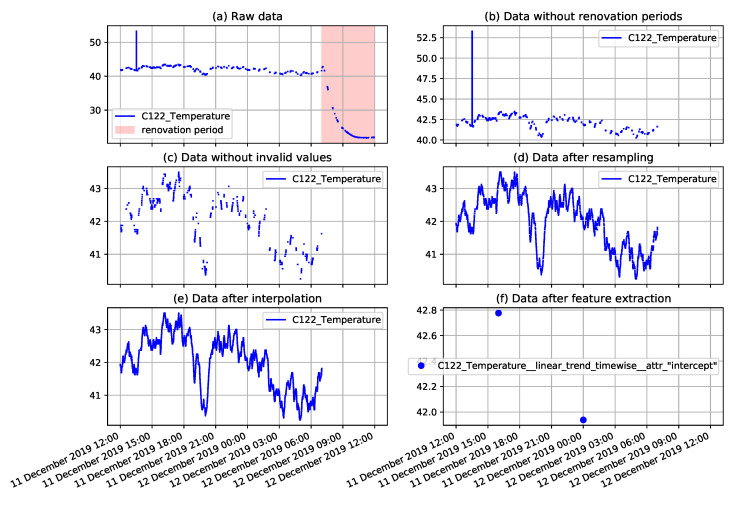
Example plots of coal crusher temperature measurements illustrating consecutive preprocessing steps including: The removal of renovation periods, removal of invalid values, resampling, interpolation, and extraction of derived features.

In case of the analysis requiring data preprocessing, the data preparation steps presented in the previous section were followed and the proper parameter values were selected. The example illustration of the preprocessing results is presented in Figure 7. The plots presented in this figure correspond to the consecutive preprocessing operations applied to the selected fragment of the temperature measurement time series collected on the NW-10 coal crusher. The applied operations include removal of renovation periods when measurements do not represent daily operation, removal of explicitly invalid values, resampling, interpolation, and finally, extraction of derived features.

Within the experiments, the quality of results depending on the size of the sliding window and feature types were verified. The size of the sliding window in which aggregation was performed was chosen from among the values in the set {4,6,8}. In the experiments, classes of derived variables were defined to verify which set of features supports the best system performance. The classes adopted include:

- *Minimal* set of features including such basic descriptive statistics as: Median, mean, standard deviation, maximum, and minimum,
- *TimeBased* set of features (derived on data having a form of time series) including such features as: Correlation coefficient, intercept of the regression line, slope of the regression line, standard error of the estimated slope (gradient), assuming normality of the residuum, the two-sided *p* value for a test whose null hypothesis is that the slope is zero, using the Wald test with *t* distribution of the test statistic.

The analysis of feature types included each of the adopted feature sets separately (*Minimal* and *TimeBased*) and their combination.

In the described use cases, the considered devices were far enough away from other stations. Therefore, the number of duplicates in the collected data set was negligible and did not require special treatment. In a situation when in intervals no measurements from a given sensor were recorded, and the break in data transmission was shorter than 50 s in case of crushers and 120 s in case of gantries, the missing data was supplemented with the last recorded value. Gaps longer than 50 s and 120 s respectively were treated as missing data. The basis for the identification of the end of the machine active periods were measurements for which the registered effective value of vibrations did not exceed a certain threshold. This threshold was set to 100 mg for coal crushers and 150 mg for gentries. The values were determined empirically. Additionally, selected values exceeding the defined threshold, but representing the return of the operating characteristics to the operating mode or a gradual fading out were deleted from the analysed data sets.

In view of the assumptions made for the proposed method, the following parameters were adopted for the performance of the base model. The initialisation of the base model is performed after collecting data representing 30 days of device operation. It was assumed that the length of the period preceding the event should be set at 2 weeks. This time should be sufficient to take preventive maintenance action. The share of outliers in the monitored data was assumed to be 1%. The base model is adapted to the new data (the new model is generated) when this threshold is exceeded. The length of the period immediately after the overhaul of the device, during which the new components run in and the data are not analysed, was set at 1 week.

In the experiments performed, several outlier identification methods were verified as a base model. The list of the methods includes most of the representatives of the approaches presented in Section 2: The HDBSCAN algorithm [48] extending the original DBSCAN method, LOF, Isolation forest, and one-class SVM. The parameter values of the base model are tuned whenever the threshold of acceptable number of identified outliers is exceeded (see Figure 1). The grid search method was applied to base model tuning and the parameters modified for each of method are presented in Table 1.

The experiments carried out included the verification of the approach with and without using the correction model. The purpose of the correction model is to learn from operator indications which alarms were false and to prevent them from being reported in the future. In the experiments presented, when the operation of the correction model was considered, it was assumed that all events occurring outside of the two-week pre-failure periods were labelled as false. The classifier performing the task of the correction model was generated using the Random Forest method. The number of trees in the ensemble was set to 40 and the maximal tree depth was set to 8.

### 5.3. Results

In the experiments, the quality of each base model was verified for each value of the selected parameters of the proposed method. Each base model was therefore run 9 times (3 values of aggregation window × 3 types of attribute set) for each set of data. Taking into account that the adopted parameters give a different representation of the data, it can be assumed that each model was run on 45 sets of data to verify the quality of the method. A summary of the results obtained in the form of mean values of the adopted quality measures (N, P, and Score) for each verified base model is presented in Table 2. The results presented in this table have been obtained using the proposed method without applying the correction model. In this way, the actual quality of the base model can be verified without the influence of the correction model, which aims to improve the quality of the system.

The results presented in Table 2 allow to determine the ranking of the base models. The ranking is based on the Score measure values. The average ranking of the base models is shown in Table 3.

The results summarised to the form of ranking in Table 3 shows that the HDBSCAN method as a base model works well for most of the problems. The results of this method are on average the best on the NW-10 and NW-20 crusher data sets, for the S202 gantry data set and synthetic data set, this model took second place. Furthermore, more detailed analysis is therefore presented for this base model.

Table 4 presents the evaluation of the proposed method with the HDBSCAN base model when the correction model was applied and the evaluation of the execution of the HDBSCAN method only. In the latter case, neither adaptation nor correction was applied in addition to the outlier detection method.

Comparing the results of the proposed method presented in Table 2 and Table 4 (without and with the correction model, respectively), it can be noticed that the correction model increases the average Score measure, decreasing the value of N and much less significantly decreasing the value of P. It shows that the correction model works as intended and reduces the number of alarms when the machine is working properly. Analysing the results of the stand-alone HDBSCAN method, it can be seen that the lack of adaptation of the base model gives very high N values especially for real data, resulting in low Score values.

Quantifying the described differences of the compared methods, the use of the base model adaptation reduced false alarms by 75.95% on average in relation to the performance of the stand-alone HDBSCAN method. The use of both adaptation of the base model and correction model reduced false alarms by 90.25% on average in relation to the performance of the stand-alone HDBSCAN method. The increase of the median Score values in these cases was equal to 35.18% and 49.73%, respectively.

**Figure 8:**
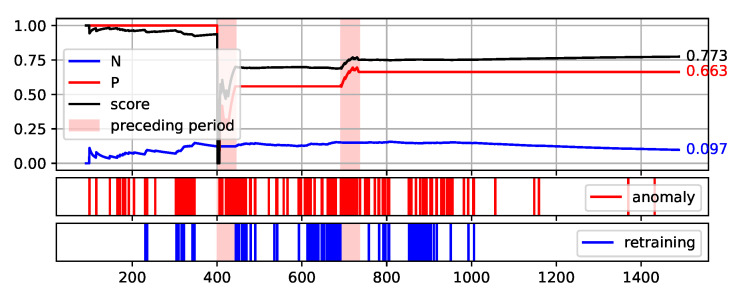
Plot of the values of each measure (P, N, and Score) over consecutive aggregation windows for HDBSCAN execution on the NW-10 data set.

**Figure 9:**
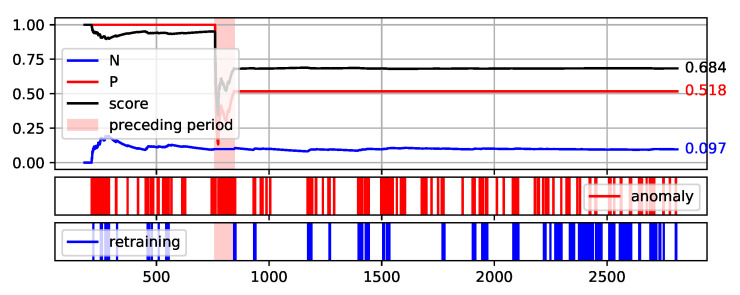
Plot of the values of each measure (P, N, and Score) over consecutive aggregation windows for HDBSCAN execution on the NW-20 data set.

**Figure 10:**
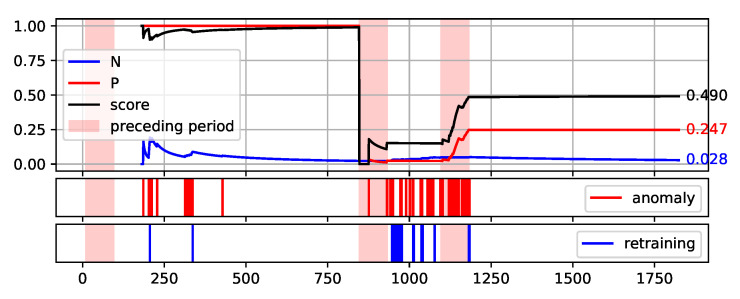
Plot of the values of each measure (P, N, and Score) over consecutive aggregation windows for HDBSCAN execution on the S201 data set.

**Figure 11:**
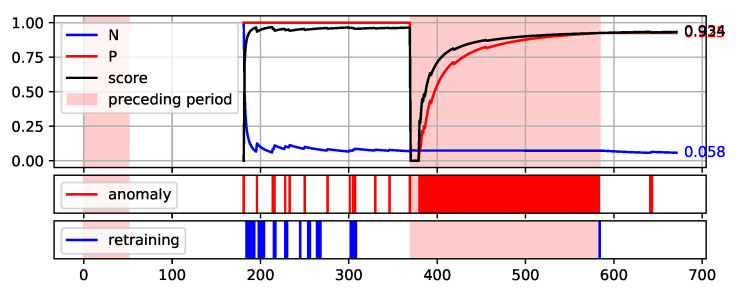
Plot of the values of each measure (P, N, and Score) over consecutive aggregation windows for HDBSCAN execution on the S202 data set.

**Figure 12:**
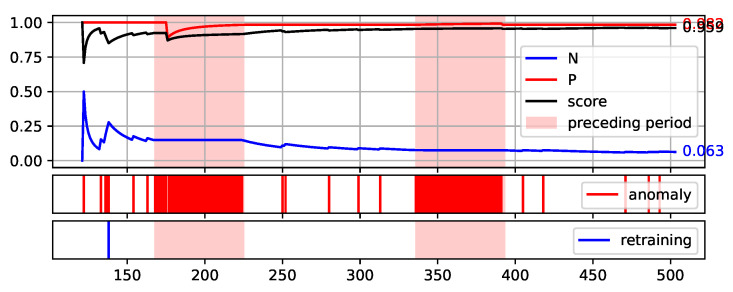
Plot of the values of each measure (P, N, and Score) over consecutive aggregation windows for HDBSCAN execution on the synthetic data set.

To fully illustrate the performance and quality of the proposed method, the plots of the values of each measure over consecutive aggregation windows are presented in Figure 8, Figure 9, Figure 10, Figure 11 and Figure 12. The bright red areas of the graphs mark the periods of two weeks prior to failure. Under each graph are marked the occurrences of anomaly identification being reported to the operator as an alarm and the occurrences of retraining the base model to maintain the assumed low number of anomalies identified.

The presented graphs were created for the best result obtained by the HDBSCAN method on each of the data sets. The values of the parameters that were adopted in obtaining the selected results and the Score value obtained are shown in Table 5.

Two of the figures presented, namely Figure 10 and Figure 12, are particularly interesting. Figure 12 illustrates the proposed method execution on synthetic data set. This data set was created to prove the correctness of the proposed method and it identifies the periods preceding the failure very well. A large number of outliers are identified during this period, reflecting the changes in data. Outside this period, the number of anomalies identified is low and there is single retraining of the base model. The analysis of data from the S201 gantry (Figure 10) is much more challenging. Three pre-failure periods are highlighted in this Figure. The first of them falls within the assumed initial period from which the data are used to initialise the models. Therefore, method predictions were not determined on this data. The second period before the failure was not identified by the method. This resulted in a significant decrease of the Score value during this period. Before the third failure, however, the method behaved properly by reporting the identification of numerous anomalies.

### 5.4. Decision Explainability

The proposed method assumes the presentation to the operator of explanations for the reported alarms related to the identification of outliers. An implementation of the SHAP method [47] was used to generate the explanations. This method determines which features had the greatest influence on the decision and which decision they supported. SHAP determines the impact of the features in relation to the base value (the average model output over the training dataset). The model decision can be an anomaly or normal value, which are represented by SHAP as −1 and 1, respectively. Therefore, the impact of the features is illustrated by the values aiming at −1 (or 1) decision, starting from the base value.

**Figure 13:**
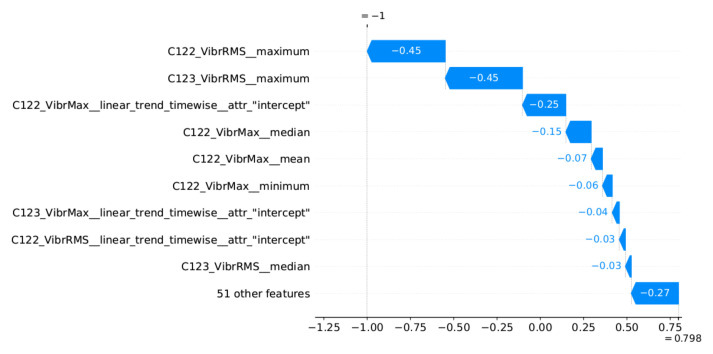
Waterfall plot of SHAP explanations for individual prediction (true positive case)—the explanation applies to the location marked by the red straight line in Figure 14.

**Figure 14:**
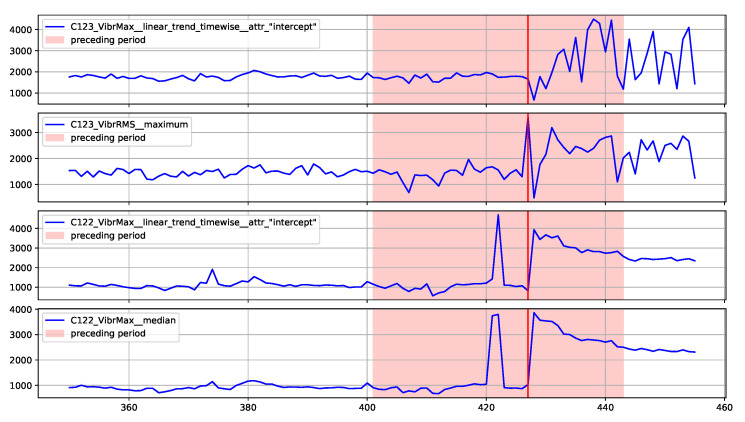
Selected excerpt from the plot of the most significant features indicated by the explanatory model in Figure 13.

**Figure 15:**
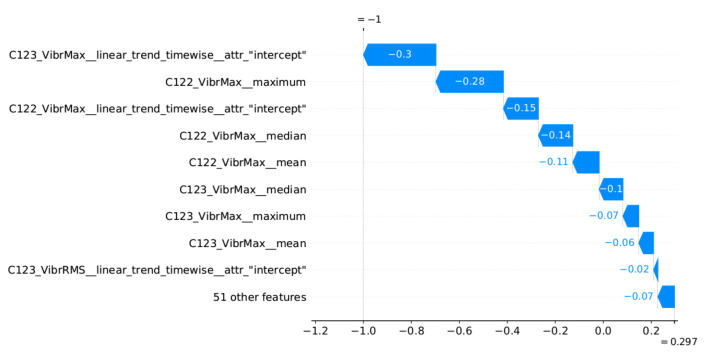
Waterfall plot of SHAP explanations for individual prediction (false positive case)—the explanation applies to the location marked by the red straight line in Figure 16.

**Figure 16:**
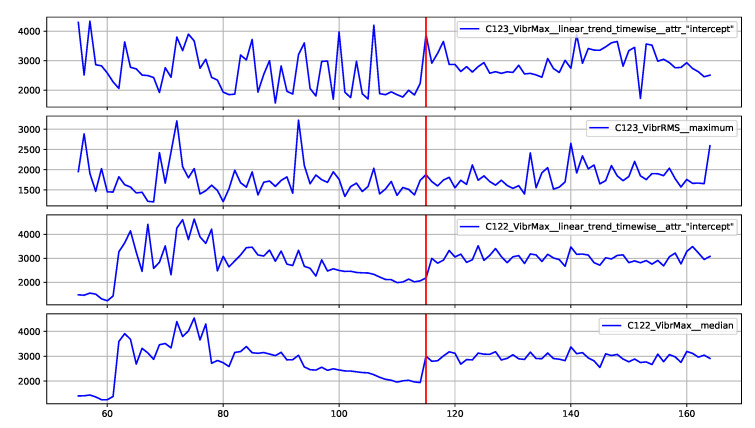
Selected excerpt from the plot of the most significant features indicated by the explanatory model in Figure 15.

Two examples of generated explanations for the NW-10 coal crusher are presented below. These explanations are supplemented with plots of feature values from the period covering the case under analysis (aggregation window). Figure 13 provides the explanation of the true positive anomaly identification case. This explanation is complemented by Figure 14 presenting the plots of the four most significant features indicated by SHAP. The explanation applies to the location marked by the red vertical line. Figure 15 provides the explanation of the false positive anomaly identification case. Again, this explanation is complemented by Figure 16, presenting the plots of the four most significant features indicated by SHAP. Explanation applies to the location marked by the red vertical line. The explanations generated in this way draw the operator’s attention to selected aspects of the measurements and can be a helpful addition to his experience in assessing the condition of the machine.

## 6. Conclusions

This paper presents a method that performs the task of predictive maintenance when the examples representing the machine failure are scarce and makes the application of supervised machine learning methods impossible. The created method operates on multidimensional time series generated from measurement data including vibration and temperature. The proposed solution is based on the detection of outliers. It assumes the interaction with the machine operator and the machine learning based correction of the system decisions in order to reduce the reported false alarms. The operator’s evaluation of the device’s state is supported by the presentation of explanations generated by XAI for decisions determined by the proposed method.

In addition to the new method, the paper proposes new measures to assess its quality. With regard to these measures, the method was verified on a synthetic data set and within two case studies evaluating its application on data from real industrial installations.

The high efficiency of the method on synthetic data indicates the correct assumptions of the proposed approach. The research carried out demonstrates the usefulness of the method and provides guidance on its configuration, such as the use of the HDBSCAN method to generate the base model. The proposed approach significantly outperformed the stand-alone outlier detection method. As a limitation of the proposed method, one can see the necessity of its initialisation, which is related to the collection of an initial data set. However, the requirements of this initialisation are minimal compared to the requirements of methods based on classification models.

The benefit for users of the proposed method will be to receive legitimate and reasoned alarms about the deteriorating condition of the machine, and thus be able to plan maintenance work. The machine operator will receive support in the form of information about alarming values of measurements. This information will not be burdensome due to the application of the false alarm reduction methods. Instead, it will be enriched by the explanations generated by the XAI, and thanks to this context, it will be more comprehensible.

Further work on the proposed method will focus on the research related to the base model generating alarm predictions. In this study, four models were verified indicating HDBSCAN as the most favourable solution. Further research may consider the implementation and verification of the other outlier detectors listed in Section 2. Moreover, the evaluation performed have shown that different methods may work best on different data. It may therefore be interesting to develop a mechanism to automatically select a method for the data being analysed. Furthermore, for some applications after a cold start period, it may be beneficial to use classification methods to identify a deteriorating machine condition. It therefore requires verification as to whether it is profitable to develop a solution that automatically switches the base model from an outlier identification method to a classification method once a sufficient number of examples representing an impending failure have been collected.

## Figures and Tables

**Table 1:** Parameters tuned for each method evaluated as the base model in the proposed approach.

Method	Parameter	Parameter Explanation
HDBSCAN	min_samples	Number of samples in a neighbourhood for a point to be considered a core point
LOF	n_neighbors	Number of neighbours to use by default for k-neighbours queries
	algorithm	Algorithm used to compute the nearest neighbours
	leaf_size	Parameter of the selected algorithms used to compute the nearest neighbours
Isolation Forest	contamination	Amount of contamination of the data set, i.e. the proportion of outliers in the data set
	bootstrap	Defines whether sampling with or without replacement should be used
One-Clas SVM	nu	Upper bound on the fraction of training errors and a lower bound of the fraction of support vectors
	kernel	Kernel type to be used in the algorithm

**Table 2:** Average results of the proposed method with respect to the base model—average of runs for different parameter values, without correction model.

		HDBSCAN	LOF	Isolation Forest	One-Class SVM
NW-10	N	0.189 (±0.022)	0.077 (±0.031)	0.098 (±0.048)	0.243 (±0.088)
	P	0.651 (±0.051)	0.115 (±0.049)	0.366 (±0.174)	0.163 (±0.039)
	Score	0.726 (±0.026)	0.318 (±0.072)	0.550 (±0.155)	0.349 (±0.044)
NW-20	N	0.317 (±0.061)	0.102 (±0.019)	0.225 (±0.096)	0.402 (±0.012)
	P	0.172 (±0.189)	0.015 (±0.013)	0.049 (±0.040)	0.042 (±0.018)
	Score	0.281 (±0.213)	0.093 (±0.072)	0.185 (±0.074)	0.156 (±0.034)
S201	N	0.046 (±0.016)	0.051 (±0.015)	0.212 (±0.028)	0.451 (±0.028)
	P	0.079 (±0.087)	0.220 (±0.135)	0.355 (±0.194)	0.658 (±0.054)
	Score	0.236 (±0.147)	0.436 (±0.145)	0.507 (±0.145)	0.600 (±0.015)
S202	N	0.310 (±0.092)	0.112 (±0.018)	0.380 (±0.065)	0.506 (±0.025)
	P	0.764 (±0.146)	0.691 (±0.138)	0.815 (±0.094)	0.670 (±0.275)
	Score	0.719 (±0.045)	0.780 (±0.080)	0.710 (±0.068)	0.562 (±0.108)
synth1	N	0.087 (±0.026)	0.018 (±0.017)	0.020 (±0.036)	0.106 (±0.098)
	P	0.934 (±0.024)	0.978 (±0.012)	0.102 (±0.104)	0.573 (±0.466)
	Score	0.923 (±0.011)	0.980 (±0.011)	0.256 (±0.195)	0.605 (±0.410)

**Table 3:** Average ranking of the base models used in the proposed method—average of runs for different parameter values, without correction model.

	HDBSCAN	LOF	Isolation Forest	One-Clas SVM
NW-10	1.00 (±0.00)	3.56 (±0.53)	2.22 (±0.44)	3.22 (±0.83)
NW-20	1.72 (±1.20)	3.28 (±1.09)	2.44 (±1.01)	2.56 (±0.73)
S201	3.89 (±0.33)	2.67 (±0.71)	2.00 (±0.87)	1.44 (±0.73)
S202	2.22 (±0.83)	1.33 (±0.71)	2.67 (±0.71)	3.78 (±0.67)
synth	2.44 (±0.53)	1.00 (±0.00)	3.67 (±0.50)	2.89 (±0.93)

**Table 4:** Average quality measures of the proposed method executed with HDBSCAN base model, adaptation of the base model and correction model, and average quality measures of the stand-alone HDBSCAN method (average of runs for different parameter values).

		Proposed Method with HDBSCAN	Stand-Alone HDBSCAN
		(With Correction and Adaptation)	(Without Correction and Adaptation)
NW-10	N	0.095 (±0.089)	0.654 (±0.082)
	P	0.602 (±0.160)	0.707 (±0.081)
	Score	0.737 (±0.379)	0.487 (±0.052)
NW-20	N	0.104 (±0.096)	0.939 (±0.077)
	P	0.161 (±0.042)	1.000 (±0.000)
	Score	0.311 (±0.191)	0.208 (±0.142)
S201	N	0.026 (±0.109)	0.903 (±0.140)
	P	0.078 (±0.459)	0.972 (±0.053)
	Score	0.237 (±0.638)	0.207 (±0.226)
S202	N	0.115 (±0.105)	0.925 (±0.039)
	P	0.737 (±0.558)	0.994 (±0.011)
	Score	0.804 (±0.677)	0.263 (±0.078)
synth	N	0.036 (±0.059)	0.457 (±0.147)
	P	0.917 (±0.567)	0.992 (±0.004)
	Score	0.940 (±0.586)	0.727 (±0.102)

**Table 5:** Parameter values adopted in obtaining the best Score value by the HDBSCAN method on each of the data sets—illustrated in Figure 8, Figure 9, Figure 10, Figure 11 and Figure 12.

	Aggregation Window	Types of Attribute Set	Score
NW-10	8	Minimal TimeBased	0.773
NW-20	4	TimeBased	0.684
S201	4	Minimal	0.490
S202	4	TimeBased	0.934
synth	6	TimeBased	0.959

## Data Availability

The synthetic data set presented in this study is openly available in: http://adaa.polsl.pl/index.php/datasets-software/ accessed on: 24 December 2021.

## Appendix A

The plot of synthetic data set is presented in Figure A1, the descriptive statistics for this data set are presented in Table A1. The plot of coal crusher data set is presented in Figure A2 containing time series for NW-10 crusher and in Figure A3 containing time series for the NW-20 crusher. The descriptive statistics for this data set are presented in Table A2. The plot of gantry data set is presented in Figure A4 containing time series for the S201 gantry and in Figure A5 containing time series for the S202 gantry. The descriptive statistics for this data set are presented in Table A3.

**Table A1:** Descriptive statistics of the synthetic data set, the statistics include: Mean value (mean), standard deviation (std dev.), minimal value (min), 25th percentile (25%), 50th percentile (50%), 75th percentile (75%), and maximal value (max).

	Mean	Std Dev.	Min	25%	50%	75%	Max
*x*1	520.78	47.14	450.00	484.42	515.82	544.11	698.48
*x*2	499.93	28.88	450.00	475.06	499.73	524.82	550.00

**Table A2:** Descriptive statistics of each attribute in the coal crusher data, the statistics include: mean value (mean), standard deviation (std dev.), minimal value (min), 25th percentile (25%), 50th percentile (50%), 75th percentile (75%), and maximal value (max).

Crusher	Sensor	Attribute	Mean	Std Dev.	Min	25%	50%	75%	Max
NW-10	C122	VibrRMS	638.85	322.14	100.00	386.00	535.00	803.00	5500.00
		VibrMax	1697.42	857.00	200.00	1018.00	1473.00	2183.00	7803.00
		Temp	34.49	4.91	3.75	31.13	34.25	37.31	56.13
	C123	VibrRMS	784.98	573.10	103.00	320.00	622.00	1034.00	4786.00
		VibrMax	2085.28	1410.79	143.00	845.00	1715.00	2916.00	7841.00
		Temp	33.84	5.69	15.38	29.75	33.25	37.69	67.75
NW-20	C124	VibrRMS	1118.55	541.05	100.00	742.00	1019.00	1362.00	6152.00
		VibrMax	3017.39	1278.28	200.00	2011.00	2995.00	3781.00	7926.00
		Temp	35.34	6.49	10.25	30.31	35.50	39.63	70.38
	C125	VibrRMS	880.50	284.61	100.00	650.00	826.00	1102.00	5280.00
		VibrMax	2349.91	761.61	181.00	1747.00	2283.00	2877.00	7855.00
		Temp	35.33	7.20	8.88	29.13	35.56	41.19	72.38

**Table A3:** Descriptive statistics of each attribute in the gantry data, the statistics include: mean value (mean), standard deviation (std dev.), minimal value (min), 25th percentile (25%), 50th percentile (50%), 75th percentile (75%), and maximal value (max).

Crusher	Sensor	Attribute	Mean	Std Dev.	Min	25%	50%	75%	Max
S201	S01	VibrRMS	287.31	380.93	4.00	28.00	39.00	454.00	3189.00
		VibrMax	636.94	724.62	32.00	90.00	133.00	1088.00	4080.00
		Temp	35.47	7.54	6.25	30.25	35.88	40.75	55.38
	S02	VibrRMS	428.90	523.57	16.00	27.00	89.00	736.00	3751.00
		VibrMax	849.30	932.22	26.00	80.00	181.00	1541.00	4080.00
		Temp	34.34	9.95	5.38	28.75	35.44	41.25	70.88
	S03	VibrRMS	296.29	402.66	16.00	27.00	71.00	451.00	3855.00
		VibrMax	618.88	746.33	26.00	80.00	160.00	981.00	4080.00
		Temp	27.19	10.03	2.19	19.19	27.88	34.50	59.06
	S04	VibrRMS	247.65	285.58	13.00	26.00	71.00	425.00	3221.00
		VibrMax	504.45	540.82	10.00	74.00	165.00	885.00	4080.00
		Temp	27.87	10.12	2.69	20.13	28.63	35.00	58.50
	S05	VibrRMS	346.17	419.20	13.00	24.00	33.00	637.00	2453.00
		VibrMax	800.73	920.28	16.00	58.00	90.00	1594.00	4080.00
		Temp	34.60	7.99	10.88	28.75	35.13	40.88	55.69
	S06	VibrRMS	449.74	528.36	15.00	26.00	87.00	888.00	3455.00
		VibrMax	899.10	984.89	10.00	80.00	181.00	1813.00	4080.00
		Temp	32.50	10.28	6.56	24.63	33.00	39.75	64.63
S202	S07	VibrRMS	324.94	412.09	12.00	26.00	95.00	502.00	3395.00
		VibrMax	747.23	884.07	16.00	80.00	197.00	1168.00	4080.00
		Temp	31.66	7.04	3.50	26.75	31.44	36.75	52.13
	S08	VibrRMS	310.33	366.67	15.00	26.00	169.00	477.00	3633.00
		VibrMax	650.31	730.05	21.00	74.00	336.00	1050.00	4080.00
		Temp	31.80	11.01	5.31	22.88	31.69	40.63	59.81
	S09	VibrRMS	126.77	132.17	18.00	30.00	70.00	177.00	2700.00
		VibrMax	288.12	294.13	21.00	96.00	160.00	378.00	4048.00
		Temp	29.58	12.31	2.50	18.69	28.50	40.00	61.06
	S10	VibrRMS	116.08	116.63	15.00	27.00	80.00	168.00	1965.00
		VibrMax	269.45	307.28	16.00	80.00	170.00	352.00	4053.00
		Temp	31.85	12.51	0.63	21.25	32.00	42.00	64.69
	S11	VibrRMS	375.41	499.33	14.00	25.00	153.00	634.00	4342.00
		VibrMax	860.60	935.34	16.00	80.00	341.00	1530.00	4080.00
		Temp	35.30	7.58	5.00	29.75	35.06	41.31	54.13
	S12	VibrRMS	312.61	382.69	18.00	29.00	137.00	499.00	3635.00
		VibrMax	673.26	736.86	37.00	96.00	298.00	1072.00	4080.00
		Temp	32.06	11.47	6.56	22.56	31.25	41.38	63.88
